# Rubeola

**Published:** 1853-10

**Authors:** 


*Rubeola.*—Dr. Walz has employed, after the manner of Schneeman, frictions with fat, in 343 cases of measles, fifty-seven of which were severe; all were cured very speedily. In thirty of these cases, the patients were tuberculous, *and the progress of phthisis was arrested.*

*Scarlatina.*—Dr. Walz has treated in the same way, seventy-four patients with scarlatina.—*Brit. and For. Med. Chir. Rev.*

